# Evaluation of a disease specific rheumatoid arthritis self-management education program, a single group repeated measures study

**DOI:** 10.1186/s12891-015-0663-6

**Published:** 2015-08-20

**Authors:** Vironique Vermaak, N. Kathy Briffa, Bob Langlands, Charles Inderjeeth, Jean McQuade

**Affiliations:** 1 School of Physiotherapy and Exercise Science, Curtin University, Perth, Western Australia Australia; 2 Department of Rheumatology, Sir Charles Gairdner Hospital, Perth, Western Australia Australia; 3 Arthritis and Osteoporosis Western Australia, Perth, Western Australia Australia; 4 School of Medicine and Pharmacology, University of Western Australia, Perth, Western Australia Australia; 5 Health, Education & Research Program Manager, Arthritis and Osteoporosis Western Australia, PO Box 34, Wembley WA 6913, 17 Lemnos St, Shenton Park, Western Australia 6008 Australia

## Abstract

**Background:**

Rheumatoid Arthritis is a progressive and disabling disease, predicted to increase in prevalence over the next 50 years. Self-management is acknowledged as an integral part in the management of chronic disease. The rheumatoid arthritis specific self-management program delivered by health professionals was developed by Arthritis Western Australia in 2006. The purpose of this study was to determine whether this program would achieve early benefits in health related outcomes, and whether these improvements would be maintained for 12 months.

**Methods:**

Individuals with rheumatoid arthritis were referred from rheumatologists. Participants with co-existing inflammatory musculoskeletal conditions were excluded. All participants completed a 6-week program. Assessments occurred at baseline (8 weeks prior to intervention), pre-intervention, post-intervention, and 6 and 12 month follow ups. Outcomes measured included pain and fatigue (numerical rating scale, 0–10), depression and anxiety (hospital anxiety and depression questionnaire), health distress, and quality of life (SF-36 version 2).

**Results:**

There were significant improvements in mean [SD] fatigue (5.7 [2.4] to 5.1 [2.6]), depression (6.3 [4.3] to 5.6 [3.9]) and SF-36 mental health (44.5 [11.1] to 46.5 [9.5]) immediately following intervention, with long term benefits for depression (6.3 [4.3] to 4.9 [3.9]), and SF-36 subscales mental health (44.5 [11.1] to 47.8 [10.9]), role emotional (41.5 [13.2] to 46.5 [11.8]), role physical (35.0 [11.0] to 40.2 [12.1]) and physical function (34.8 [11.5] to 38.6 [10.7]).

**Conclusion:**

Participants in the program recorded significant improvements in depression and mental health post-intervention, which were maintained to 12 months follow up.

## Background

Arthritis is one of the most prevalent chronic diseases in Australia [1], with nearly 0.5 million affected by the most severe type, rheumatoid arthritis (RA) [2]. Rheumatoid arthritis is an autoimmune disease of unknown aetiology [3, 4]. More women are affected by RA than men with 2.6 % and 1.6 % of the Australian population respectively affected [5]. By 2050, it is projected to affect 0.9 million Australians [1].

Rheumatoid arthritis causes progressive deterioration, with subsequent functional decline [6], leading to disability, participation restrictions and impaired quality of life [7]. Individuals with RA experience greater psychological distress than the general population [8]. Furthermore, over 80 % of individuals with RA have clinically important fatigue [9]. Pain, a cardinal symptom of RA, affects up to 84 % of individuals [10], and negatively impacts on multiple aspects of life [11].

Self-management (SM) is increasingly being accepted as an integral part in the management of chronic disease [12]. Self-management interventions (SMIs) are patient-centred, problem focused and action-oriented [13, 14], addressing physical and psychosocial issues [15]. They utilise educational, behavioural and cognitive strategies [12, 14] to enhance patient participation in treatment [16].

Education programs (EPs) teaching SM skills are believed to be more effective than information-only EPs in improving clinical outcomes in RA patients [17, 18]. However, reported benefits are equivocal and the benefits are temporary [17]. Systematic reviews have reported a trend towards small benefits from arthritis SM programs [16, 19–21]. Overall, SMIs improve knowledge, SM skills, self-efficacy (SE), and some aspects of health status [22, 23]. There is evidence supporting positive outcomes for disease-specific SMIs [16, 19, 20], including osteoarthritis [24]. Numerous studies have evaluated EPs and generic SMIs, however, only a small number have examined disease-specific SMIs. Disease-specific SMI are reported to produce better outcomes compared to generalised non-specific chronic disease SMIs [25, 26], and it is thought that this approach may be more beneficial for people with RA. The available evidence suggests that provision of tailor-made programs may be more beneficial for chronic disease management [27, 28] and thus identification of individual educational needs becomes imperative [29].

The RA SMI was developed by Arthritis Western Australia in 2006, using a participatory action research model, utilising the Plan, Do, Study, Act approach. The program was based on the findings of an educational needs assessment tool survey completed by a total of 157 RA patients from United Kingdom (n = 125) and Western Australia (n = 32) in 2005 [30]. Findings demonstrated that people with RA have specific needs [30]. It was on this basis, that the RA SMI was developed. Participant needs catered for incorporated offering the program in a community setting with flexible delivery times including evenings and weekends as well as the content of the program. The intervention program is person-centred and based on SM principles, including cognitive behavioural therapy and SE constructs.

### Purpose of study

The aim of this study was to determine whether the RA SMI would achieve immediate improvements in health related outcomes following program involvement, and whether these improvements would be maintained for 12 months after course completion.

## Methods

### Study design

A one group (within subjects) repeated measures study was used to determine change over time.

This study was approved by the Human Research Ethics Committee at Curtin University, Western Australia (PT0021, 2005). All participants provided written informed consent.

### Participants

Patients with RA were recruited by referral from specialist rheumatologists in Western Australian public hospitals and private sectors. Inclusion criteria were clinical diagnosis of RA made by their treating rheumatologist, aged ≥18 years, and the ability to speak and understand English. Participants with co-existing inflammatory musculoskeletal conditions were excluded. Diagnostic criteria were not specified but at the discretion of the clinical rheumatologist treating the patient.

### Outcome measures

Outcome measures evaluated pain, fatigue, depression, anxiety, SE, health distress and health related quality of life. Participants completed a set of baseline (BL) questionnaires 8 weeks prior to the commencement of the program , then again one week prior to the first SM session (pre-intervention), one week after the 6 week program (post-intervention), with follow up periods at 6 and 12 months.

*Pain and Fatigue* were each measured via a ten-point incremental numerical rating scale. Numerical rating scales have adequate discriminative power for describing pain intensity [31], have good sensitivity [32] and the reliability is excellent (r = 0.94) [33].

*Depression and Anxiety* were measured using the 14-item Hospital Anxiety and Depression scale, consisting of 7 anxiety and 7 depression items, scored separately [34] which is reliable (Cronbach’s alpha 0.68 to 0.93) and valid [34, 35].

*Self-Efficacy* was measured using an 8-item questionnaire developed by Stanford University from the original 20-item arthritis SE scale [36]. The scale reflects how participants rate their certainty in performing tasks along a continuum. This scale is reliable and valid [36, 37] (http://patienteducation.stanford.edu/research/searthritis.html).

*Health Distress* was measured using a 4 item questionnaire to assess the level of fear, worry and/or frustration each individual felt in relation to their disease [37]. Scores range from 0 to 5 with higher scores indicating more distress about health (http://patienteducation.stanford.edu/research/searthritis.html).

*Quality of life* was measured using the Short-Form 36 Version 2 questionnaire (SF-36). The SF-36 consists of 36 questions and includes 8 component subscales reflecting physical and mental status [38], including physical function, role physical, bodily pain, general health, vitality, social function, role emotional and mental health. The SF-36 is a well-established outcome assessment in RA [39, 40] and is reliable and valid [41–43].

### Intervention

The newly developed RA SMI program was offered to patients managed in a Rheumatology Outpatient Department of a tertiary hospital and in a community setting. Groups of 8–15 participants attended 1 session each week for 6 consecutive weeks, with each 2.5 h in duration. Attendance and assessment time-points were recorded. Modules are inter-related and cumulative building on previously learned information. The program was delivered by the same two health professionals. Uniformity of the RA program across and within groups was maintained by using scripted facilitator’s manual.

The program content concentrated on disease-specific education including:

- Psychological impact and management strategies
- Fatigue management and energy conservation strategies
- Pain management
- Medications
- Exercise
- Posture, balance and falls prevention
- Nutrition
- Complimentary/alternative therapies
- Joint protection
- Osteoporosis

Personal development focused on individual weekly goal setting, problem solving, relaxation techniques, and cognitive behavioural therapy to assist in long term behavioural changes. Participants were encouraged to incorporate techniques learnt at each session into their activities of daily living, returning to discuss their experience next week.

### Data analysis

Data was analysed using SPSS version 19. One way (repeated measures) analysis of variance (ANOVA) with time (baseline, pre-intervention, post-intervention, 6 and 12 months follow up) as the independent variable was utilised. Mauchly’s Test of Sphericity was performed on all data. When the Mauchly’s statistic was significant, with the sphericity assumption violated for the univariate approach to repeated-measures ANOVA, this was corrected with the Greenhouse-Geisser adjustment. Where tests of within-subjects comparisons were significant, implying a significant change over time, post-hoc linear contrasts were performed to compare each time point to the pre-intervention time point. Significant improvements between BL and pre-intervention were interpreted to indicate spontaneous improvement, suggesting that changes following the intervention may also be spontaneous. Statistical significance was inferred at p < 0.05.

## Results

A total of 113 participants (96 [85 %] female) with a mean (SD) age of 54 (13) were enrolled in the study. Ninety-six participants completed data collection at all time-points. Of these participants 92 (96 %) attended 80 % or more of the intervention sessions.

All patients in the study were referred by and being treated by a clinical Rheumatologist at either a public hospital or private clinic. Treatment prescribed by these specialist physicians was at their discretion in consultation with the patient taking into account efficacy, tolerance, toxicity, cost, compliance and patient preference. At baseline, 43 %, 76 % and 13 % of participants were taking steroids, disease modifying anti-rheumatic drugs (DMARDs) and biologics (bDMARDs), respectively. Steroid use decreased to 35 % post-intervention and 32 % at 12 months. DMARDs use increased to 79 % at pre-intervention but stabilised at 76 % post-intervention and at 12 months. The use of bDMARDs continually increased from 15 % pre-intervention to 16 % post-intervention and 24 % at 12 months. At 12 months, 59 % of participants were taking symptomatic treatments such as paracetamol and NSAIDS.

### Fatigue and pain

Fatigue and pain remained stable during the pre-intervention control period. The fatigue numerical rating score decreased significantly from a mean (SD) of 5.7 (2.4) pre-intervention to 5.1 (2.6) post-intervention, however, there was a small rebound to 5.2 (2.7) at 6 and 12 months which was no longer statistically significant (Table 1). Pain numerical rating score showed a small non-significant decrease throughout the course of the study.

**Table 1:** Results for outcomes: fatigue, pain, anxiety, depression, health distress, self-efficacy, and SF-36 at baseline, pre-intervention, post-intervention, 6- and 12-months follow up. Data are mean (SD)

Outcomes	Baseline	Pre-Ix	Post-Ix	6 Months	12 Months
Fatigue (0–10)	6.1 (2.4)	5.7 (2.4)	5.1 (2.6)^a^	5.2 (2.7)	5.2 (2.9)
Pain (0–10)	5.1 (2.4)	4.9 (2.2)	4.8 (2.7)	4.9 (2.7)	4.4 (2.9)
Anxiety (0–21)	7.8 (4.1)	7.4 (3.9)	6.9 (3.9)	6.7 (4.0)^a^	6.7 (4.5)
Depression (0–21)	6.1 (3.8)	6.3 (4.3)	5.6 (3.9)^a^	5.2 (3.8)^a^	4.9 (3.9)^a^
Health distress (0–5)	2.3 (1.1)^a^	2.1 (1.1)	2.0 (1.0)	1.7 (1.2)^a^	1.4 (1.1)^a^
Self-efficacy (0–10)	5.5 (2.1)^a^	5.9 (1.9)	6.1 (2.1)	6.1 (2.3)	5.9 (2.8)
*SF-36 (0–100)*					
Mental health	44.1 (10.6)	44.5 (11.1)	46.5 (9.5)^a^	47.2 (10.6)^a^	47.8 (10.9)^a^
Role emotional	40.5 (13.4)	41.5 (13.2)	42.6 (13.4)	45.3 (13.1)^a^	46.5 (11.8)^a^
Social function	39.6 (12.3)^a^	41.5 (12.1)	42.1 (11.1)	42.8 (13.1)	43.3 (11.9)
Vitality	38.8 (10.3)^a^	40.9 (10.9)	41.7 (10.4)	43.7 (11.5)^a^	43.7 (12.2)^a^
General health	39.3 (10.9)	40.2 (11.5)	39.3 (11.3)	41.0 (11.6)	41.5 (11.5)
Bodily pain	37.3 (7.2)^a^	38.9 (7.6)	38.8 (7.5)	39.8 (9.0)	40.6 (9.4)
Role physical	32.7 (10.2)^a^	35.0 (11.0)	36.2 (12.1)	39.3 (13.0)^a^	40.2 (12.1)^a^
Physical function	34.6 (11.0)	34.8 (11.5)	35.6 (11.3)	37.3 (11.4)^a^	38.6 (10.7)^a^

^a^significantly different to pre-intervention (p < 0.05). Repeated measures ANOVA

Decreasing scores in fatigue, pain, anxiety, depression and health distress signify improvement, while increasing scores in self-efficacy and SF-36 signify improvement

*Ix* Intervention

### Anxiety and depression

Levels of anxiety and depression remained stable during the control period. Anxiety had a non-significant reduction from a mean (SD) of 7.4 (3.9) to 6.9 (3.9) during the 6 week intervention. At 6 months, anxiety had decreased to 6.7 (4.0) and this level was maintained at 6.7 (4.5) at 12 months. Depression improved significantly post-intervention and was maintained to 12 months (Table 1).

### Health distress

Health distress lessened significantly during the pre-intervention control period. Post-intervention, only a small non-significant reduction was observed from mean (SD) 2.1 (1.1) to 2.0 (1.0). Further significant improvements in health distress were observed at 6 and 12 month (Table 1).

### Self-efficacy

During the pre-intervention period, a significant improvement in SE was observed, with no incremental significant changes post-intervention.

### Quality of life

The SF-36 subscales of mental health, role emotional, general health and physical function remained unchanged during the pre-intervention control period, while social function, vitality, bodily pain and role physical demonstrated significant improvements. Only mental health showed a significant improvement immediately post-intervention and mental health remained significantly better than pre-intervention at both 6 and 12 months. Although there were only small non-significant improvements in role emotional and physical function at post-intervention, improvements continued over time and were statistically significant by 6 and 12 months (Table 1).

## Discussion

This, one of the first studies of SM specifically for people with RA delivered by health professionals, demonstrated significant improvements in some, but not all, aspects of health and well-being measured. This results appears superior to those from non-disease specific SM programs, however, participants with RA included in generic SM arthritis studies are usually in the minority [23, 25, 44, 45], consequently presenting limitations to interpreting their results for this subset of participants. It is not clearly understood how SMIs affect change in health outcomes. Several factors that may have an affect are motivation to participate and to change health behaviour, deteriorating health, stressful life events [28], the concept of readiness to change [46, 47], and lower quality of life [40]*.* Our results highlight that some changes may only develop in the long term. For example improvements in anxiety, role emotional and physical function were only evident at 6 months.

Improvements in anxiety, depression and mental health are important due to their severe impact. Participants were taught how to manage the psychological impacts of RA through relaxation and distraction techniques. Improving these SM skills should enable better coping with the challenges imposed by RA [48–50]. Better knowledge and understanding of disease empowers and enhances a sense of control, and consequently people feel less anxious [7]. Armed with better behavioural and cognitive strategies to manage their disease, they are likely to be more confident, and feel less uncertain about the consequences of their disease, [48, 51], with associated improvements in mental health and depression [49, 52, 53]. Our results reflect this as participants reported experiencing fewer role limitations due to emotional problems. Barlow et al. [44] found similar improvement in anxiety and depression following participation in the Arthritis Self-Management Program (ASMP). However, no immediate post-intervention results were recorded, and results were for the combined arthritis population, therefore making it difficult to distinguish effects within the RA participants.

There was a significant improvement in health distress during the pre-intervention control period, therefore results need to be interpreted with caution. The improvement during the control period could be attributed to the participants’ knowledge that they were joining the SMI in the near future and looking forward to a supportive environment with qualified health professionals providing reassurance. However, as for anxiety, depression and mental health, it is plausible that a greater knowledge and understanding of disease gained during the SMI, in addition to SM strategies, could reduce fear, worry and/or frustration felt in relation to RA. Other studies evaluating the effect of ASMP in an arthritis population support our findings [23, 25, 45]. Interestingly, when Lorig et al. [23] evaluated the effect on the RA population alone, no significant effect was found. Evidence from our study suggests participation in a disease-specific SMI reduces the distress associated with RA for up to 1 year.

During the program, participants were provided with relaxation and energy conservation and pacing strategies to assist with managing fatigue. It is plausible that this SMI assisted participants to establish a balance between rest and exercise resulting in overall improvements in fatigue. In addition, participants also learned to recognise fear-avoidant behaviour, which can affect physical functioning. Instead they were able to implement alternative approaches to manage activities. Fatigue is influenced by perceptions of RA and sense of control over the consequences [54]. Therefore, altering people’s perceptions may subsequently mediate improvement in fatigue [49]. Other studies have reported conflicting results for improvement in fatigue. Following participation in the ASMP the reported improvement was maintained for 1 year [23, 25, 46]. However, this was not true for the RA population alone [23].

This intervention was designed to enhance patients’ abilities to control their pain, and to utilise adaptive pain management strategies. However no significant reduction in pain was recorded. This finding is not unexpected. Effective pain management can be difficult to achieve in patients with RA as it relates to inflammation rather than mechanical factors. Strategies to supress inflammation, especially pharmacological, would be the most effective. Our findings are consistent with previous studies that report that many patients with RA believe their pain is uncontrollable [14], and isolated SMI may not have been sufficiently potent to change this. Moreover, pain often persisted regardless of concurrent treatment and co-management with their usual clinician based care [7, 10]. Integration of a SM program into usual clinician based care as opposed to parallel delivery has been shown to have a positive impact on adherence to medical therapy and disease activity scores [55]. As we did not measure either of these outcomes in our study we cannot comment definitively on whether lack of improvement in disease activity may have been associated with the limited improvements in pain and fatigue observed in our study.

Changes in SE regarding pain and other symptoms relate to changes in perceived health status [56]. In people with RA there is a gradual deterioration in physical function [6, 57] as perpetuated by disease progression leading to disability, and impaired quality of life [58]. Patients with RA often consider their health as deteriorating, and this may be the reason for the report of no improvements in general health in the study.

Self-management interventions are often associated with improvements in SE. This is true for our study, however the improvement was not statistically significant [23, 25, 46]. Although the study used feedback questionnaires in the program to further optimise SE, there are other factors that could have affected the results [59]. Firstly the age and disease duration of the hospital cohort may have an influence as they had long disease duration and severe deformities [60]. Also the participants may have set unachievable goals and unrealistic expectations, and the subsequent failure weakened SE [14, 61]. The small but insignificant improvement in SE is consistent with findings following an online ASMP [23]. This study’s findings are also consistent with the previously reported negative association between pain ratings and perceived SE [60].

As this was a SMI study, medical interventions were not part of the assessment brief. This was managed in parallel by independent clinicians without specific knowledge of the SMI interventions and based on their independent individual patient assessments. Ongoing medical management is an important confounding variable that we were unable to control for in this study. Given the progressive destructive nature of the disease, we did not consider it ethical to withhold medical revision during the 12 month study period. Changes in medication have the potential to influence a number of the variables measured. This may in part explain the findings of significantly improved health distress, SE, bodily pain and role physical during the control period. It is plausible that better controlled pain improves SE, minimising perceived role limitations due to physical functioning, and reduced health distress. Use of DMARDS did not change overall, however bDMARDS did and may have a significant impact on outcome measures. Biologics are considered the ‘ideal’ and the most effective treatment in combination with a DMARD in terms of disease control and or remission. Therefore the increase in prescription rates may be considered a strength of this program as it encouraged more optimal treatment. This may be a consequence of better education and empowerment of participants. Improving education and self-efficacy in conjunction with optimisation of medical management to reduce symptoms of inflammation and signs of destruction would be desirable to optimise patient outcomes.

One limitation of the study is the design. A single group repeated measures design was used to evaluate short and long term benefits, so all participants could partake in the intervention. The effect of time and “any contact” with any care provider is difficult to factor in or correct for. Nevertheless, longitudinal and observational studies provide essential information about the course and outcome of rheumatic diseases that cannot be provided by randomised controlled trials [62]. However, to further develop and examine the effectiveness of the RA SMI a large randomised controlled trial would be required.

Findings from this study are important for implementing future RA SMIs. These interventions are relatively inexpensive and safe forms of treatment. They also support current health care funder priorities of shifting the management of chronic diseases into the primary care environment and the emphasis on person centred management with less reliance on specialist centres.

## Conclusions

In conclusion implementing a RA SMI demonstrated significant benefits. Any contact with a supportive health care provider with a specific disease focus, appears to be beneficial for patients with RA. There appears to be additional benefit of adding health professional intervention and SMI. Improvements in depression and mental health were observed in response to the SMI. Moreover, they were maintained at 12 months. The reported increase in prescription of bDMARDS by patients’ medical practitioner should be viewed as a positive and essential adjunctive strategy for disease modification and reducing impairment and disability. Empowering patients through education may allow them to be more proactive in seeking better evidence based medical treatments earlier. As this was one of the first studies on RA SMI future development and evaluation via a randomised controlled trial is necessary to continue to optimise the positive outcomes of the program. In particular refinement of the program focussing on the SE and pain outcomes needs to be planned, implemented and evaluated.

## Abbreviations

RA: Rheumatoid arthritis
SM: Self-management
SMI: Self-management intervention
EP: Education program
SE: Self-efficacy
SF-36: Short form 36
ANOVA: Analysis of variance
DMARD: Disease modifying anti rheumatic drug
bDMARD: Biologics
ASMP: Arthritis self-management program
